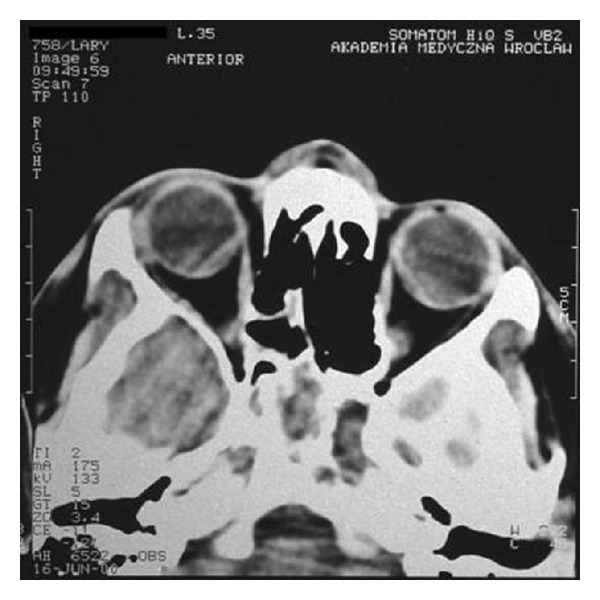# Ameloblastoma of the Nasal Septum Origin: A Case Report

**DOI:** 10.1155/2013/280509

**Published:** 2013-09-21

**Authors:** Hanna Temporale, Tomasz Zatoński, Anna Roszkowska, Tomasz Kręcicki

**Affiliations:** Department of Otolaryngology Head and Neck Surgery, Wroclaw Medical University, Borowska 213, 50-556 Wrocław, Poland

## Abstract

*Background*. Ameloblastoma is the most common odontogenic tumor. It represents about 1% of all tumors of the jaw. Extragnathic location of the ameloblastoma is typical and extremely rare. *Case Report*. We report a case of ameloblastoma of the nasal septum origin, causing nasal obstruction. According to our information, this is the first reported case of ameloblastoma coming from the nasal septum as a primary tumor without maxillary sinus involvement. *Conclusions*. Ameloblastoma can not only locate in the maxilla and mandible, but also in other regions of the craniofacial. Ameloblastoma should be considered in the differential diagnosis of tumors causing nasal obstruction. Nonspecific clinical features of sinonasal ameloblastoma make it extremely important to perform accurate diagnostic imaging and histopathological examination.

## 1. Introduction

Ameloblastoma is the most common odontogenic tumor. It represents about 1% of all tumors of the jaws [1]. It occurs four times more often in the mandible than in the maxilla; a variety of peripheral locations are also listed. Ameloblastoma is a benign epithelial tumor, derived from the residual dental lamina or from the developing enamel organ. This neoplasm usually does not cause pain and grows slowly, causing the distension of the bone. Ameloblastoma may be locally invasive—can invade the surrounding tissues—giving frequent recurrence after nonradical surgery (45–90%). Rare forms of malignant ameloblastoma-carcinoma ameloblasticum can occur. Then it may give distant metastases to the lungs, lymph nodes, and spine [2, 3].

Macroscopic appearance of ameloblastoma is usually a cavernous tumor, cushioned with a thin lining, sometimes without tumor capsule, filled with gray-white or gray-pink gelatinous mass. The microscopic examination reveals focuses of epithelial tissue and mimicking enamel organ, surrounded by mature connective tissue rich in collagen.

The tumor shows a considerable variety in radiological imaging—presenting a view of one or more multi-cave cysts—sometimes containing a primordial tooth or images of soap bubbles, honeycomb, or solid tumor appearance [4].

Usually ameloblastoma is diagnosed late, in advanced stages, due to its long and scantily-symptomatic development. Wide surgical excision of the tumor in the normal tissue margin is considered to be the treatment method of choice.

Ameloblastomas developed in the maxilla can secondarily grow in the nasal cavity and paranasal sinuses, but the primary focus of ameloblastoma in this area, with no relation to the jaw area, is extremely rare. There are only a few reports of such cases in the literature [1, 5–7].

We report a case of ameloblastoma of the nasal septum origin, causing nasal obstruction. According to our information, this is the first reported case of ameloblastoma coming from the nasal septum as a primary tumor without maxillary sinus involvement. The previous reports of ameloblastomas causing nasal blockade were associated with cases of tumors expanding into the nasal cavity from a starting point in the maxilla.

## 2. Case

A thirty-five-year-old man, had undergone septoplasty in the Department of Plastic Surgery because of impaired nasal patency lasting for three months. Due to the lack of improvement reported 3 years after surgery, he was admitted to the district ENT ward. The rhinoscopy showed complete obstruction of the right nasal passage due to creation of a cyst, extending from the nasal septum. The cyst was removed surgically. The histopathological examination revealed the cyst wall fragment with chronic inflammation.

**Figure 1:**
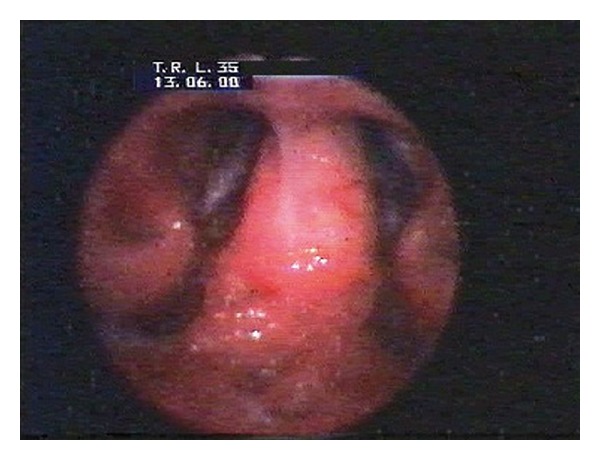


**Figure 2:**
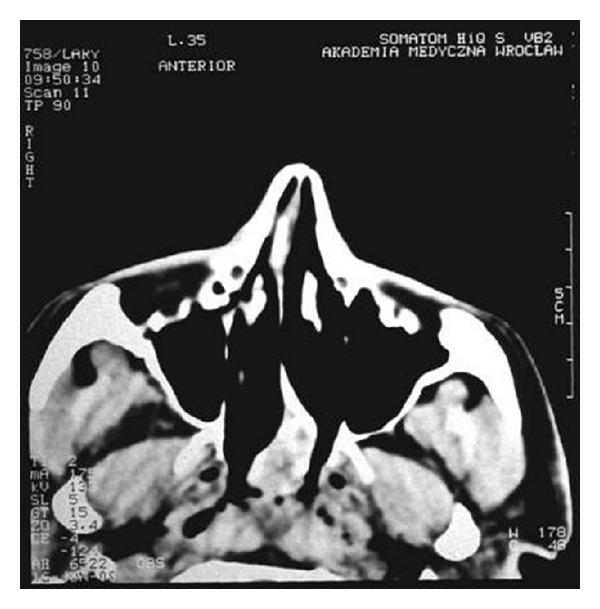


**Figure 3:**
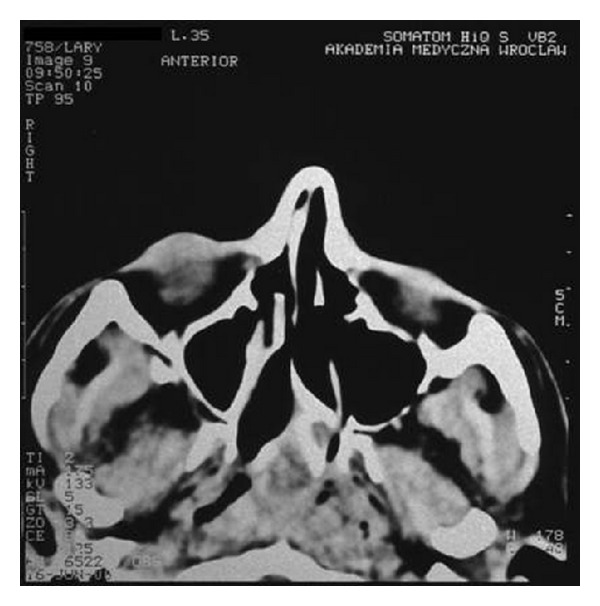


**Figure 4:**
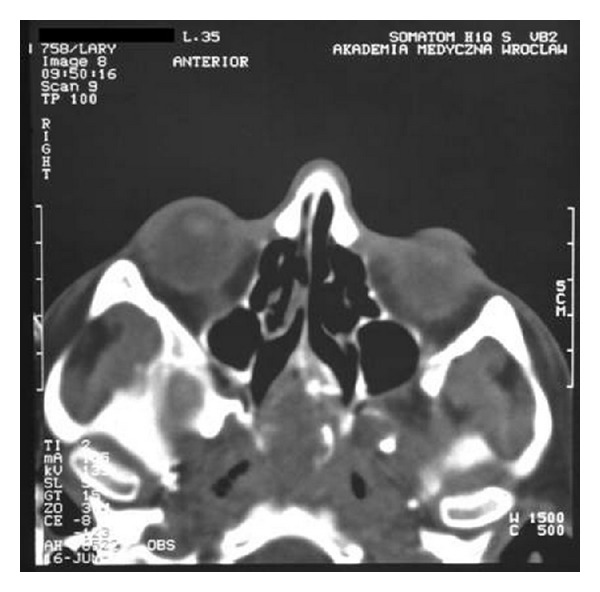


Nasal obstruction appeared again after two months. The another biopsy of lesions was collected for histopathological examination. The specimen has been described as ameloblastoma. The patient was transferred to the Department of Otolaryngology Head and Neck Surgery, Medical University of Wroclaw. On admission, the patient's main complaint was nasal obstruction on the right side. In the posterior rhinoscopy, a tumor of the posterior upper part of the nasal septum was revealed (Figure 1). The computed tomography scans showed a soft tissue mass involving nasopharynx, ethmoid, and sphenoid sinus (Figures 2, 3, 4, and 5).

The tumor was removed surgically. For the radical surgery and in the interest of cosmetics, the access through eversion of the face coverings (“midfacial degloving”) was chosen. The removal of the front and medial wall of the right maxillary sinus with lower concha allowed an easy access to the tumor occupying the upper part of the posterior nasal septum and passing to the sphenoid sinus. The advantage of this method is obtaining no external cuts and, consequently, a good aesthetic result. The tumor was completely removed. There was no tumor in the right maxillary sinus and nasopharynx. The tissue samples from the tumor margin were collected.

The histopathological examination confirmed the diagnosis of ameloblastoma. The margins of the removed tumor were free of tumor cells. The postoperative course was uncomplicated. The patient after surgery feels good. Nasal patency is still correct and he remains under constant care of ENT outpatient clinic.

## 3. Discussion

Extragnathic location of the ameloblastoma is unusual and extremely rare [6, 7]. The peak incidence of ameloblastomas usually falls on the third decade of life, with no gender predilection. In comparison to the ameloblastomas of the jaws, it is believed, however, that those located within the sinuses and nasal cavities are more common in older men [5, 6].

The diagnosis of the sinonasal tumors at an early stage is difficult. It is important to collect the interview carefully, pay attention to the symptoms of a unilateral nature, and perform physical examination necessarily accompanied by nasal and nasopharynx endoscopy. Ameloblastoma diagnosis is based on the clinical and radiological image with the decisive histopathological confirmation. However, the differential diagnosis can cause problems, especially during the examination of a biopsy of the small fragments of tissues or obtained superficially, due to the lack of assessment of the whole structure of the tumor [5, 6]. Radiologically, the standards of diagnosis of ameloblastomas are computed tomography scans and magnetic resonance imaging [8].

The ameloblastomas differential diagnosis of the sinonasal region takes into account the most common acute and chronic sinusitis, inverted papilloma, squamous cell carcinoma, adenocarcinoma, angiofibroma, basal cell adenoma, and proliferation of craniopharyngioma [6]. In the case of lesions located in the nasal, septum congenital or acquired pathologies can be suspected. Congenital anomalies of the nasal septum are rare (encefalocele, glioma, teratoma). The acquired pathologies of the septum may be caused by trauma, infection, toxic damage (cocaine), inflammation, or tumors (primary and secondary). Inflammatory diseases which may occupy the nasal septum include Wegener's granulomatosis and sarcoidosis. Tumors that can occur in this location are squamous cell carcinoma, adenocarcinoma, cystic epidermal carcinoma, adenoid-cystic carcinoma, melanoma, sarcoma, esthesioneuroblastoma, chloroma, lymphoma, plasmacytoma, Pindborg tumor, schwannoma, angiofibroma, hemangioma, and metastases [9].

The treatment of choice of ameloblastomas and other sinonasal tumors is surgery. In some cases, the endoscopic procedures are applied [5, 6]. The prognosis of the treatment depends mainly on the tumor size and surrounding tissue attachment as well as the resection radicality [5]. Relapses can occur even after a period of 15 years, that is why there is a special emphasis on the long-term postoperative patient control [6, 7].

## 4. Conclusions

This case shows that ameloblastoma can be located not only in the maxilla and mandible but also in other regions of craniofacial. Ameloblastoma should be considered in the differential diagnosis of tumors causing nasal obstruction. Nonspecific clinical features of sinonasal ameloblastoma make it extremely important to perform accurate diagnostic imaging and histopathological examination.

Despite the seemingly benign tumor histology, the lesions should be removed *en bloc* with a wide margin. The implementation of a fast, radical surgical treatment in the highly-specialised center is a key for the successful prognosis and for avoiding complications. A long-term followup is required after the completion of the treatment [4].

## Figures and Tables

**Figure 5:**